# A tunable acoustic metamaterial with double-negativity driven by electromagnets

**DOI:** 10.1038/srep30254

**Published:** 2016-07-22

**Authors:** Zhe Chen, Cheng Xue, Li Fan, Shu-yi Zhang, Xiao-juan Li, Hui Zhang, Jin Ding

**Affiliations:** 1 Lab of Modern Acoustics, Institute of Acoustics, Nanjing University, Nanjing, 210093, P. R. China

## Abstract

With the advance of the research on acoustic metamaterials, the limits of passive metamaterials have been observed, which prompts the studies concerning actively tunable metamaterials with adjustable characteristic frequency bands. In this work, we present a tunable acoustic metamaterial with double-negativity composed of periodical membranes and side holes, in which the double-negativity pass band can be controlled by an external direct-current voltage. The tension and stiffness of the periodically arranged membranes are actively controlled by electromagnets producing additional stresses, and thus, the transmission and phase velocity of the metamaterial can be adjusted by the driving voltage of the electromagnets. It is demonstrated that a tiny direct-current voltage of 6V can arise a shift of double-negativity pass band by 40% bandwidth, which exhibits that it is an easily controlled and highly tunable acoustic metamaterial, and furthermore, the metamaterial marginally causes electromagnetic interference to the surroundings.

Currently, acoustic metamaterials are extensively studied owing to unprecedented characteristics, which exhibit application potentials in various fields^1^,^2^,^3^,^4^,^5^,^6^. However, with the development of acoustic metamaterials, the limitations of passive metamaterials have been observed. First, the characteristic frequency bands related to extraordinary acoustic performance are restricted into narrow bands induced by resonance. In addition, since the unique characteristics of metamaterials are created by artificial structures, they cannot be freely changed once the metamaterials are fabricated. Therefore, tunable acoustic metamaterials with adjustable characteristic frequency bands are urgently required treading on the heels of their counterparts in electromagnetics and optics^7^,^8^,^9^,^10^. Different mechanisms were adopted to realize tunable metamaterials. First, tunable features arose from nonlinear effects in acoustic metamaterials, in which the constitutive parameters could be changed with the intensities of input acoustic fields, and they were considered to be self-modulation metamaterials^11^,^12^,^13^. On the other hand, actively tunable metamaterials, which could be controlled by an external source other than input acoustic fields, were presented. As an example, by mechanically changing the volumes of Helmholtz resonators in a metamaterial, the resonant frequency was adjusted, which could change the corresponding characteristic frequency bands^11^,^14^. A similar method on the analogy of the split-ring resonators in electromagnetic metamaterials was introduced into acoustic metamaterials, in which the resonant frequency of a split hollow sphere was tuned by filling water into the sphere to change the volume^15^. Similarly, the equivalent sound speed could be changed by filling a fluid into the intermediate gaps of a metamaterial^16^. Generally, an external electric signal is preferred as the control signal in tuning the performance of a metamaterial. Piezoelectric materials, which can convert an electric signal into an acoustic vibration, were adopted to create actively tunable acoustic metamaterials. First, piezoelectric disks were introduced into one-dimensional metamaterial on the basis of the elements of membranes^17^,^18^ or Helmholtz resonators^19^,^20^. Through a feedback loop system, the effective dynamic densities or modulus of the metamaterials could be adjusted, resulting in variable transmissions and characteristic frequency bands. Furthermore, an actively controlled acoustic cavity by piezoelectric materials was used in an adjustable metamaterial for realizing negative refraction and imaging^21^. Then, an tunable acoustic metafluid was created based on a piezoelectric transducer driven by the signals controlled by a sensor detecting the acoustic waves incident on the metamaterial, which was a feedback system realizing an adjustable effective mass density, modulus and transmission^22^. Meanwhile, a similar mechanism was adopted to create a non-reciprocal and highly nonlinear metamaterial^23^ and a superlens reconfigurable in real time overcoming the diffraction limit of a linear lens^24^. Magnetic fields were also used to adjust the performance of an acoustic metamaterial, in which the alinement of discoid microparticles was controlled by an external magnetic field determining the elastic constants and acoustic speed in the metamaterial^25^.

Recently, elastic membranes were widely applied in the design of acoustic metamaterials. First, a negative density was produced in membrane-type metamaterials with a single membrane^26^ or periodically distributed membranes^27^. Then, double-negativity was achieved on the basis of two types of scatterers, membranes and side holes^28^,^29^. Furthermore, membrane-type metamaterials could be used to realize zero mass^30^, sound insulation and absorption^31^,^32^,^33^,^34^. Therefore, the studies on actively adjustable metamaterials on the basis of membranes have been given consideration^35^. A special membrane made of magnetorheological material was set up in a magnetic field with gradient intensity^36^, and then the resonant frequency of the membrane was changed because the magnetorheological material can react to the external gradient magnetic field, allowing for an adjustable effective density and transmission in a low-frequency range. Besides, a membrane coated with a gold layer was set up in an extremely strong electric field up to 1000 V. Electric field forces were exerted on the gold-coated membrane, and the performance of the membrane-type metamaterial was controlled by the strong external electric field^37^.

In this work, we design a tunable acoustic metamaterial with double-negativity on the basis of a passive metamaterial composed of periodically arranged side holes and ordinary elastic membranes^28^,^29^. The effective stiffness of the membranes is controlled by externally exerted mechanical stresses produced by direct-current (DC) electromagnets. By changing the DC voltage supplied to the electromagnets, the double-negativity pass band (DPB) and the phase velocity in the metamaterial are controlled and adjusted. Experiments exhibit that a tiny DC voltage of 6 V can shift the DPB by 40% of the bandwidth and induce considerable variation in negative phase velocity. Via the electromagnets converting an external electric voltage into stiffness variation in the elastic membranes, this metamaterial can be easily and conveniently controlled and adjusted to a great extent merely with a low-voltage DC source. The adjusting of the metamaterial requires no special equipment and no treating on the membranes, and thus, the presented mechanism for controlling a metamaterial is available in various membrane-based metamaterials.

## Results

### The band edges determined by the scatterers in a double-negativity metamaterial

**Figure 1:**
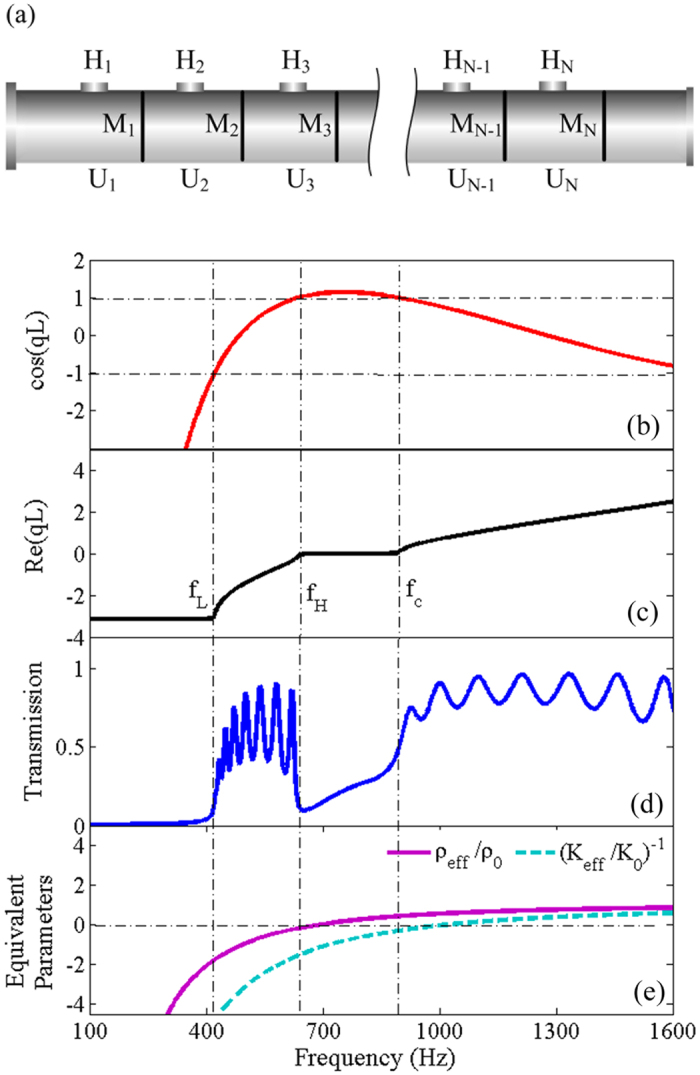
Model and simulated performance of a passive metamaterial. (**a**) Model of a passive metamaterial with double-negativity based on periodically distributed membranes and side holes along a pipe. (**b**) cos (*qL*), (**c**) dispersion Re(*qL*), (**d**) transmission spectra and (**e**) equivalent density and modulus of the tunable metamaterial before the electromagnets are electrified, which are calculated with the parameters: a unit length *L* = 10 *cm*, a radius of main pipe *a* = 1.3 *cm*, a radius of side hole *r* = 1.1 *cm*, a mass of membrane *m*_*M*_ = 3.78 *mg*, a stiffness of membrane *k*_*M*_ = 1275 *N*/*m*, a unit number *N* = 10, and the parameters of air *ρ*_0_ = 1.21 *kg*/*m*^3^ and *c*_0_ = 344 *m*/*s*.

Figure 1a shows the model of a passive metamaterial with double-negativity based on periodically distributed membranes (*M*_*i*_) and side holes (*H*_*i*_) along a pipe (*i* = 1, 2, 3… *N*), which act as two types of scatterers^13^,^28^. The metamaterial is divided into units (*U*_*i*_) and the dispersion can be expressed to be^13^:


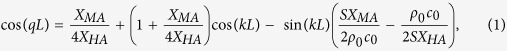


in which *q* is the wave number of the Bloch wave and *L* is the length of the unit. *ρ*_0_ and *c*_0_ are the density and acoustic velocity of the air in the metamaterial, respectively. *k* and *ω* = *c*_0_*k* are conventional wave number and circular frequency, respectively. According to Bloch theory and plasma model^28^,^38^,^39^, a membrane is considered to be an acoustic reactance 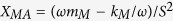, where *S* = *πa*^2^ and *m*_*M*_ are the area and mass of the membrane, respectively. *k*_*M*_ is the effective stiffness of the membrane equal to 8*πF*_*τ*_, where *F*_*τ*_ is the tension in the membrane. A side hole can be seen as an acoustic impedance *Z*_*HA*_ = *R*_*HA*_ + *jX*_*HA*_, where 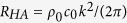 and 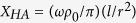 are the effective acoustic resistance and reactance, respectively. *r* and *l* are the radius and effective length of the side hole, including the additional length induced by the acoustic radiation^38^. According to the impedance relations at the positions of the scatterers, the input impedance and transmission coefficient of the metamaterial can be obtained by successively using the impedance transfer equation^13^,^39^.

Figure 1b–d show the dispersion and transmission spectra of the metamaterial with ten units when no controlling signal is supplied. It can be observed that there is a low-frequency forbidden band below the frequency *f*_*L*_ = 420 Hz, and then, a pass band arises with the lower and upper cut-off frequencies of *f*_*L*_ and *f*_*H*_ = 650 Hz, respectively. Finally, behind a forbidden band from *f*_*H*_ to *f*_*c*_ = 890 Hz, the other pass band occurs.

To tune the characteristic bands of the metamaterial, we must determine the relationship between the band edges and the scatterers. First, according to Bloch theory, *f*_*L*_ is characterized by cos (*qL*) = −1 and *f*_*H*_ and *f*_*c*_ are characterized by cos (*qL*) = 1, as shown in Fig. 1b. Then, from equation (1), it can be proven that the cut-off frequencies *f*_*H*_ and *f*_*c*_ in the presented metamaterial, in which the membranes and side holes are symmetrically distributed, are identical to those in the metamaterial solely composed of periodic membranes or side holes^13^ (See Methods). Additionally, *f*_*H*_ and *f*_*c*_ are respectively determined by 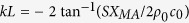 and 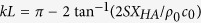, which are independently related to the membranes and side holes, respectively, and the cut-off frequency *f*_*L*_ is related to both membranes and side holes^13^. Furthermore, using plasma theory, the equivalent density and modulus can be calculated with the same equations for the membrane-based^27^ or side-hole-based metamaterials^40^, which are: 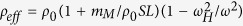 and 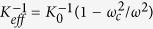, respectively^28^, where 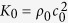 is the modulus of the air in the pipe, 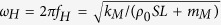 and 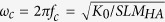, with 
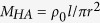
. As indicated by the equivalent density and modulus in Fig. 1e, when *f* > *f*_*c*_, the pass band originates from double-positivity, when *f*_*H*_ < *f* < *f*_*c*_, the forbidden band is induced by a negative modulus, and when *f* < *f*_*H*_, the pass band exhibit double-negativity. However, it must be noted that the effective-medium theory only makes sense under the condition of long-wavelength approximation when the lattice constant *L* is much smaller than the effective wavelength in the metamaterial, thus around the cut-off frequencies *f*_*H*_, where the effective wavelength approaches infinity^29^,^41^. Outside the frequency range, a meaningful effective-medium description is unavailable and cannot be used to explicate the occurrence of the forbidden band below *f*_*L*_. According to Bloch theory, the low-frequency forbidden band is reasonable because the dispersion equation cos (*qL*) < −1 when *f* < *f*_*L*_, as shown in Fig. 1b,c. Besides, according to the equivalent circuit theory, the metamaterial can be modeled as a composite right/left handed transmission line^29^,^41^. At low frequencies, the equivalent circuit describing the coupling of both scatterers is dominated by a series capacitance determined by the membrane and a shunt inductance determined by the side hole, which forms a high-pass filter exhibiting the left-handed behavior and *f*_*L*_ is the cut-off frequency indicating the lower edge of the left-handed behavior^29^,^41^. On the other hand, in the view of acoustic transmission, the input impedance of the metamaterial can also be obtained from the equivalent circuit, which shows that below the cut-off frequency *f*_*L*_, the input resistance of the metamaterial is zero^41^, demonstrating that no acoustic energy transmits through the metamaterial. Therefore, it can be observed that the pass band between *f*_*L*_ and *f*_*H*_ is a characteristic pass band in this metamaterial which exhibits negative density and negative modulus simultaneously.

### Tuning the metamaterial with electromagnets

Since the characteristic pass bands and unique acoustic effects in the metamaterial are induced by two scatterers, membranes and side holes, we can tune the metamaterial by adjusting the parameters of the scatterers. The parameters and performance of the side holes cannot be easily changed, while the performance of the membranes is adjustable. If the effective stiffness *k*_*M*_, namely the impedance *X*_*MA*_, of the membranes is changed, *f*_*L*_ and *f*_*H*_ can be adjusted accordingly, which provides a possibility of tuning the DPB of the metamaterial.

**Figure 2:**
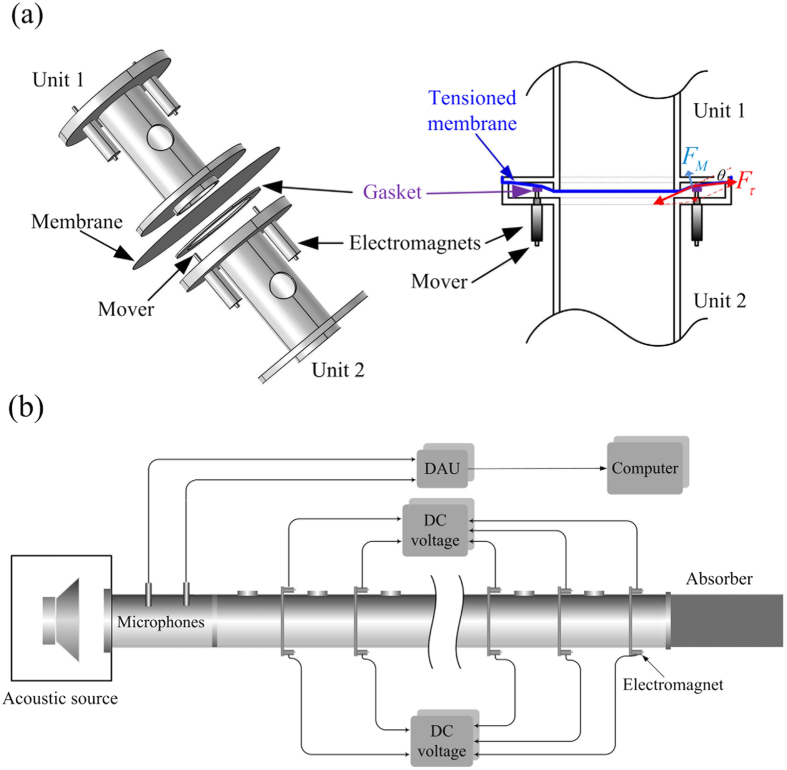
Structure of tunable metamaterial and experimental apparatus. (**a**) Schematic of the special structure for controlling and adjusting the effective stiffness of the membranes with electromagnets. (**b**) Experimental apparatus for measuring the transmission of the tunable double-negativity metamaterial with a two-microphone method.

To control the performance of the membranes, a special structure is designed to fix the membranes between adjacent units of the metamaterial, which is exhibited in Fig. 2a. A membrane covers the opening of one unit, where the margin of the pipe slightly stretches out of the flange. A thin and light circular gasket is set up around the pipe opening and is pressed onto the stretched membrane by two electromagnets. The stators of the electromagnets are fixed on the flange of the adjacent unit, and the movers touch the circular gasket and exert a force on the membrane. By supplying voltage to the electromagnets, the movers are promoted by the electromagnetic force and press the circular gasket, which produce additional tension in the membrane and change the performance of the membrane.

Figure 2b shows the experimental apparatus to measure the transmission of the tunable metamaterial with double-negativity, in which the structural parameters are the same as those used for the theoretical calculation in Fig. 1. The transmission of the active metamaterial is achieved with a two-microphone method^42^,^43^, by which the transmission spectrum in a wide frequency range can be directly obtained from a single measurement via an excitation of a wideband pulse or white noise. The electromagnets controlling the membranes are parallel connected to a DC voltage source, and by changing the driving DC voltage supplied to the electromagnets, the performance under different driving voltages can be measured with the apparatus.

**Figure 3:**
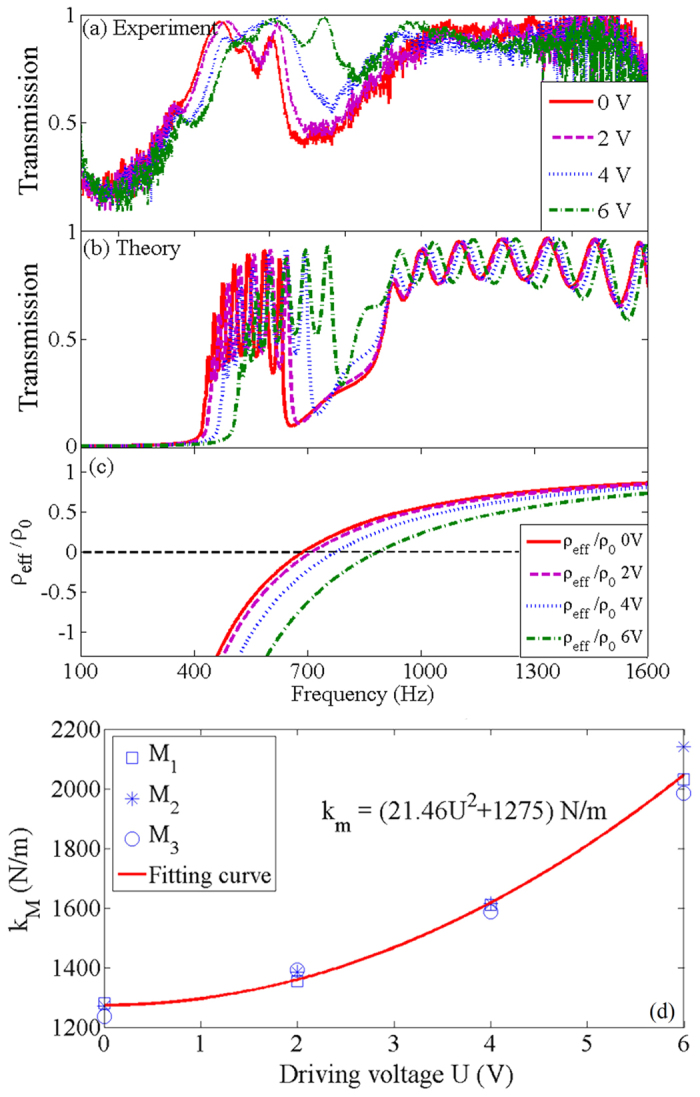
Experimental demonstration of tunable double-negativity pass band. (**a**) Experimental, (**b**) theoretical transmissions and (**c**) simulated equivalent density of the tunable double-negativity metamaterial under different driving voltages supplied to the electromagnets. They all demonstrate that the DPB is shifted to higher frequencies when the driving voltage is increased. (**d**) Variation of the effective stiffness of the membranes versus the driving voltage: M_1_, M_2_ and M_3_ are measured stiffnesses and the solid line indicates a fitting result.

Figure 3a shows the measured transmissions of the metamaterial when the electromagnets are driven by a DC voltage varying from 0 to 6 V. It can be observed that with the increase of the driving voltage, the DPB of the metamaterial is shifted to higher frequencies. Under a 6 V DC voltage, the cut-off frequency *f*_*L*_, which is related to both the membranes and side holes, is increased by 50 Hz, and *f*_*H*_, which is solely determined by the membranes, is increased by 130 Hz. Thus, the central frequency of the DPB is shifted from 535 Hz to 625 Hz, which is 40% of the bandwidth. Therefore, it is demonstrated that the metamaterial is highly tunable and sensitive to the controlling voltages. Because the tuning of the metamaterial requires no special equipment and no treating of the membranes, the controlling mechanism is available in various membrane-based metamaterials. Furthermore, since the metamaterial can be easily and conveniently controlled with a tiny DC voltage, it marginally causes electromagnetic interference to the surroundings.

The shift of the DPB in the metamaterial can be explicated by the variation of the effective stiffness *k*_*M*_ of the membranes, which originates from the additional tension in the membranes induced by the electromagnets. Figure 3d indicates the variation of *k*_*M*_ with the driving voltage *U* supplied to the electromagnets, which shows that *k*_*M*_ is quadratically related to *U*. The stiffness of the membrane is measured with an apparatus similar to that used in a passive metamaterial^13^,^27^. It is shown that *k*_*M*_ increases from 1275 N/m to 2047 N/m when the driving voltage increases from 0 to 6 V, which demonstrated that a external voltage of 6 V can increase the stiffness of the membranes by 62%. As shown in Fig. 2a, the movers of the electromagnets are attached on the circular gasket which presses the membrane onto the opening of the pipe. When the electromagnets are electrified, the movers are pushed by the electromagnetic force while they cannot move due to the block of the gasket and the membrane. Therefore, as indicated in Fig. 2a, the driving force exerted on the mover is amplified and converted into the tension in the membrane, which increases the effective stiffness of the membrane.

According to the Maxwell’s electromagnetic force equation, the force induced by a DC electromagnet can be expressed by 
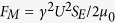
, where *S*_*E*_ is a cross-sectional area, *μ*_0_ is a magnetic permeability, *B* is a magnetic flux density and *γ* = *B*/*U* is a coefficient between *B* and the driving voltage *U*. Figure 2a shows that the tension *F*_*τ*_ in the membrane and the force *F*_*M*_ exerted on the membrane by the electromagnet exhibit a relation of 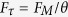, in which the angle *θ* results from slight deformation of the membrane caused by *F*_*M*_. Because *θ* is extremely small, the tension *F*_*τ*_ is much larger than *F*_*M*_, and a small variation Δ*F*_*M*_ can produce a considerable change in *F*_*τ*_. Therefore, a tiny DC voltage can effectively increases the tension in the membrane. Additionally, the effective stiffness *k*_*M*_ of the membrane is directly proportional to the tension *F*_*τ*_ with a coefficient of 8*π*^27^, and thus, *k*_*M*_ can be expressed by an equation 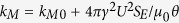, where *k*_*M*0_ is a stiffness of the membrane when *U* = 0. It can be observed that *k*_*M*_ is quadratically related to the voltage *U*, which is in accordance to the experimental results shown in Fig. 3d, in which an equation 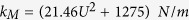 is obtained from the fitting curve. According to the measured effective stiffness *k*_*M*_, the theoretical transmissions and equivalent density of the metamaterial under different driving voltages are achieved, as shown in Fig. 3b,c. It can be observed that the theoretical and experimental results are in basically agreement with each other, and they demonstrate that the DPB is shifted to higher frequencies when the driving voltage is increased. Furthermore, comparing the calculated and measured transmissions, it can be found that the measured transmission in the DPB is slightly higher and more flat than the calculated result, which is induced by the experimental system. First, in the calculation, the effective impedance of the side hole is considered to be 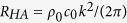 and 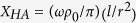, in which the resistance *R*_*HA*_ is taken as the radiation resistance of a hole set up on a flat and large baffle. While in the experiments, the side holes are set up along a cylindrical tube. As a result, the real radiation resistance of the side hole is smaller than that in the theoretical model, which results in a higher measured transmission. Additionally, the fluctuations in the pass band are produced by the coupling between the units in the metamaterial. In the tunable metamaterial, a special structure is adopted to adjust the stiffness of the membrane. As shown in Fig. 2a, due to the pipe margin slightly stretching out of the flange for fixing the membrane, a small interval arises between adjacent units. Therefore, the coupling between the units is weakened and the pass band in the tunable metamaterial is more flat than that obtained in a passive metamaterial^13^,^28^.

**Figure 4:**
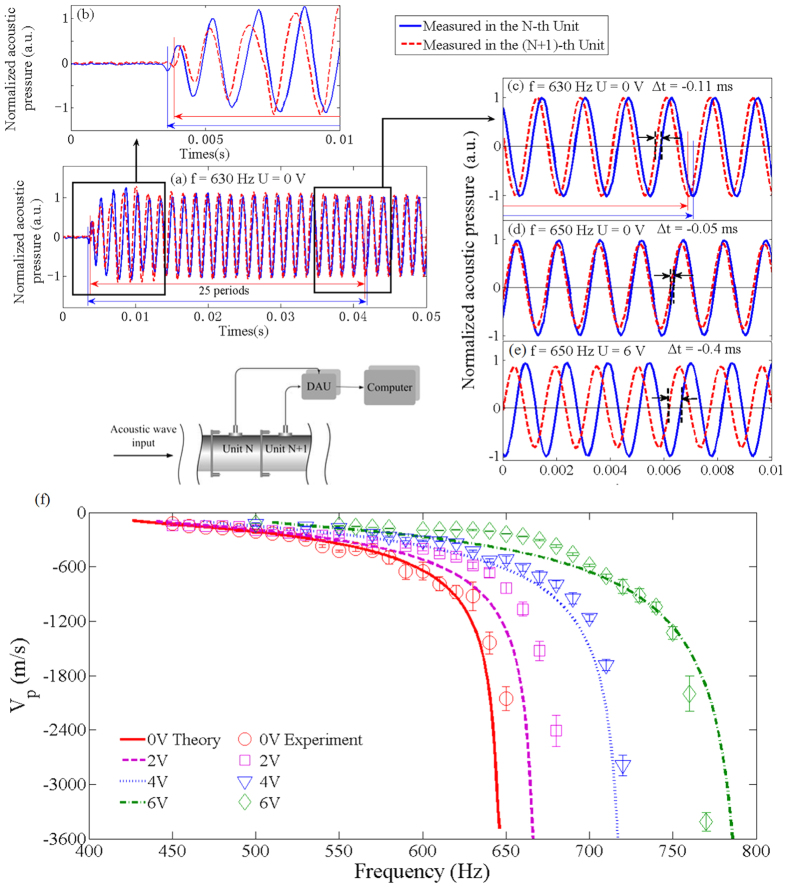
Measurements of phase velocities tunable metamaterial. (**a**) Quasi-sinusoidal signals containing the wave fronts measured in the N-th (solid line) and (N + 1)-th (dashed line) units at 630 Hz; (**b**,**c**) zoom-in of the wave fronts and the zero-crossing points obtained in the twenty-fifth periods of the signals from (**a**,**d**) detected signals at 650 HZ with the driving voltage of 0 V; (**e**) detected signals at 650 Hz with the driving voltage of 6 V. (**f**) Theoretical and experimental phase velocities in the DPB under different driving voltages supplied to the electromagnets.

Furthermore, time-domain acoustic signals in adjacent units of the metamaterial are measured with two microphones separated by 10.8 *cm* to evaluate the phase velocity in the DPB, as indicated in the inset of Fig. 4. The phase velocity can be obtained from the time difference Δ*t* between the acoustic signals measured in units N and (N + 1). In the experiments, we adopt a quasi-sinusoidal signal, which contains the wave front, as the excitation. Then, as indicated in Fig. 4a–e, the time difference Δ*t* between the detected signals in units N and (N + 1) is achieved by comparing the zero-crossing points in the same period (the 25th period) counted from the wave fronts of both signals. Using this method, the uncertainty in the measuring of the phase velocity induced by the periodicity of a sinusoidal signal can be eliminated. Figure 4c–e show that the phase of the acoustic wave measured in unit (N + 1) leads that in unit N, which exhibits that the metamaterial has a negative phase velocity in the DPB. In Fig. 4(c), the time difference at 630 Hz is Δ*t* = −0.11 *ms*, exhibiting a phase velocity of *v*_*ph*_ = −945 *m*/*s*. At a higher frequency 650 Hz closer to the upper cut-off frequency of the DPB, Δ*t* changes to −0.05 *ms*, which indicates a negative phase velocity *v*_*ph*_ = −2160 *m*/*s* with a larger absolute value. In addition, the measured acoustic signals at 650 Hz under different driving voltages are compared in Fig. 4d,e. When the driving voltage increases form 0 to 6 V, the time difference between both signals is increased from −0.05 *ms* to −0.4 *ms*, which shows that the phase velocity decreases from −2160 *m*/*s* to −270 *m*/*s*. Meanwhile, the equivalent phase velocity in the DPB can be calculated with Bloch wave-number *v*_*p*_ = *ω*/*q* or the plasma model^29^ 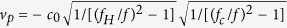. Figure 4f shows that the theoretical phase velocities and the measured results are in agreement with each other. It can be seen that the absolute values and dispersion of the negative phase velocity considerably increase when the frequency approaches the upper cut-off frequency of the DPB. When the driving voltage for the electromagnets increases, the cut-off frequencies of the DPB are increased, which is in accordance to the results indicated by the transmission. Thus, at a determined frequency *f*, the absolute value of phase velocity, which is directly proportional to 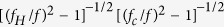, is decreased accordingly.

### Transient responses of the tunable metamaterial

**Figure 5:**
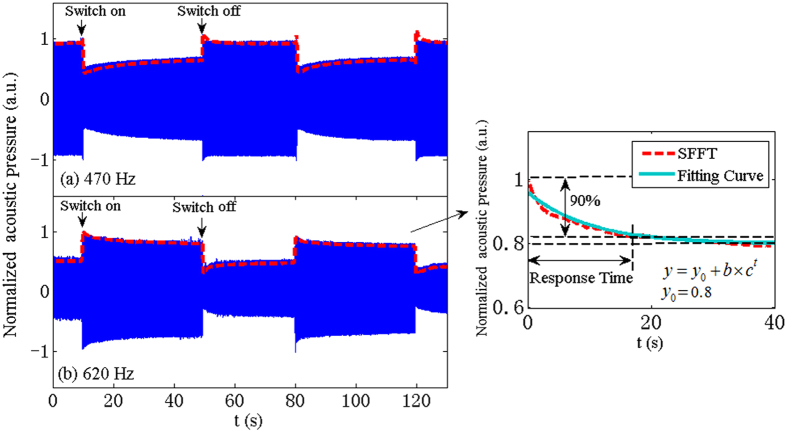
Transient responses of tunable metamaterial. Normalized acoustic signals measured in the third unit *U*_3_ at (**a**) 470 Hzand (**b**) 620 Hz when the driving voltage switches between 0 V and 6 V. The envelops of the amplitudes are extracted with STFT, which are indicated by red dashed lines. The inset shows the definition of the response or recovery time of the tunable metamaterial.

To determine the response time to the switching of the controlling voltage, the transient responses of the tunable metamaterial are measured, as shown in Fig. 5. Continuous sinusoidal acoustic waves are excited by the loudspeaker and the acoustic pressures are measured with a microphone set up in the third unit *U*_3_. Two measuring frequencies of 470 Hz and 620 Hz are chosen, when no driving voltage is supported to the system, both frequencies locate near the lower and upper band edges of the DPB, respectively. As indicated in Fig. 5a,b, at 470 Hz, the measured acoustic pressure decreases when the driving voltage is switched on because the frequency shifts from the DPB to the forbidden band, while at 620 Hz, the measured acoustic pressure increases after exerting the driving voltage because the frequency is shifted to the DPB. Then, after we turn off the driving voltage source, the acoustic pressure restores to the original value. Delays can be observed in the responses of the acoustic pressures to the switching of the controlling voltage. When the electromagnets are electrified, they exert forces onto the membrane which instantly produce a large deformation in the membrane. Because the restoring force induced by the deformation is temporarily larger than that produced by the electromagnets, the deformation recovers slightly until the tension of the membrane and the electromagnetic force come to a balance. Then, the system results in a steady state and the acoustic pressure stays constant. When the driving voltage is switched off, the electromagnetic force exerted by the electromagnets disappears, and thus, the deformation of the membrane gradually recovers and the stiffness of the membrane returns to the original value. The response and recovery times can be respectively defined to be the periods taken by the metamaterial to return to the steady states from the jumping when the driving voltage is switched on and off. Table 1 exhibits the response and recovery times of the metamaterial driven by different voltages, which are achieved from the transient responses shown in Fig. 5 using Short-time Fourier Transform (STFT). It is reasonable that a higher driving voltage, inducing a larger deformation in the membrane, results in a longer response time. However, it can be observed that the recovery time marginally varies with the driving voltage because once the extra forces induced by the electromagnets are removed, the recovery of the membrane deformation is primarily determined by the elasticity of the membrane.

## Discussion

In summary, we create a tunable acoustic metamaterial with double-negativity in which electromagnets are adopted to control the effective stiffness of the membranes, and thus, the DPB and the phase velocity of the metamaterial can be adjusted. By virtue of a large tension enhancement in the membranes induced by the electromagnets, the DPB of the metamaterial can be considerably changed with a tiny DC voltage driving the electromagnets. Theoretical and experimental results demonstrate that the DPB is shifted to high frequencies by increasing the driving voltages of the electromagnets, and the phase velocity in the DPB can be changed accordingly. Thus, the metamaterial can be easily and highly adjusted with a low-voltage DC source, which requires no special equipment and no treatment on the elastic membranes and marginally exerts electromagnetic interference to the surroundings. Therefore, the mechanism presented for adjusting the characteristic frequency band of the metamaterial can be adopted in any membrane-based metamaterials, which exhibits a great potential for the application in various fields requiring wide-band or adjustable acoustic metamaterials and vulnerable to electromagnetic interference, such as ultrasonic diagnosis and treatment, tunable filters, better negative-refraction imaging devices and other reconfigurable acoustic devices.

## Methods

### Numerical simulations

The numerical simulations are carried out on the basis of Bloch theory, impedance transfer equation and plasma model, in which the parameters adopted in the simulations are the same as those in the experiments: a unit length *L* = 10 *cm*, a radius of main pipe *a* = 1.3 *cm*, a radius of side hole *r* = 1.1 *cm*, a mass of membrane *m*_*M*_ = 3.78 *mg*, a stiffness of membrane *k*_*M*_ = 1275 *N*/*m*, a unit number *N* = 10, and the parameters of air 
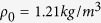
 and 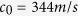. In Fig. 3, the stiffness of membrane *k*_*M*_ increases from 1275 N/m to 2047 N/m when the driving voltage increases from 0 to 6 V.

The relations between the cut-off frequencies *f*_*H*_ and *f*_*c*_ and the scatterers can be obtained from the dispersion equation (1). First, we rewrite equation (1) into:


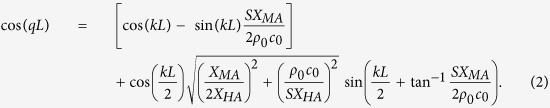


The items in the first square bracket of equation (2) just make up the dispersion equation of the negative-density metamaterial based on periodical membranes^27^. Then, the cut-off frequency *f*_*H*_ for the low-frequency forbidden band in the negative-density metamaterial^27^ is determined by 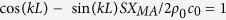, which can be rewritten as 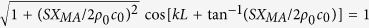. Because *kL* > 0 and *X*_*MA*_ < 0 in the low-frequency range, solving the equation, we have 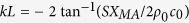 at the cut-off frequency *f*_*H*_. In this case, the second part in equation (2) equals 0, which makes equation (2) equal 1. Therefore, the dispersion equations (1) for the metamaterials with double-negativity also equals 1 at *f*_*H*_, which demonstrates that the cut-off frequencies *f*_*H*_ in both metamaterials are the same.

Similarly, we can also rewrite the dispersion equation (1) to be:


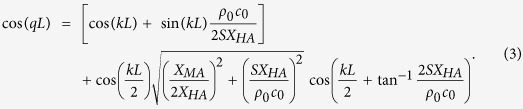


The items in the first square bracket of equation (3) just make up the dispersion equation of the negative-modulus metamaterial based on periodical side holes^40^, which equals 1 at the cut-off frequency *f*_*c*_ determined by 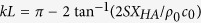. Meanwhile, it can be obtained that the dispersion (3) of the double-negativity metmaterial equals 1 at *f*_*c*_, and thus, the cut-off frequency *f*_*c*_ of the double-negative metamaterial is in accordance to that of the negative-modulus metamaterial.

Therefore, it is proven that the cut-off frequencies *f*_*H*_ and *f*_*c*_ in the double-negativity metamaterial are respectively and independently determined by the membranes and side holes. In the metamaterial studied in this work, we have *f*_*H*_ < *f*_*c*_ and the DPB locates between *f*_*L*_ and *f*_*H*_, while if we adjust the parameters of the scatterers to obtain *f*_*c*_ < *f*_*H*_, the DPB locates between *f*_*L*_ and *f*_*c*_.

### Experimental apparatus

The apparatus for measuring the performance of the metamaterial is shown in Fig. 2b. A loudspeaker (Beyma, 10MI100) is adopted to input acoustic waves into the metamaterial. The loudspeaker is enclosed by a two-layer box to reduce the influences of the acoustic waves outside the metamaterial. Two mini microphones with the diameters of 6 mm (B&K 2670) are set up at the input of the metamaterial. The signals obtained with both microphones are collected by a data acquisition unit (DAU) and input into a computer. Then, the transmission of the metamaterial is obtained by two-microphone method. According to the principle of two-microphone method, the auto-spectral densities of the incident (*S*_*ii*_) and reflected (*S*_*rr*_) acoustic waves at the input of the metamaterial can be separated using the signals obtained by both microphones, regardless of the boundary condition at the end of the metamaterial. Then, via the excitation of a wideband pulse or white noise, the transmission spectrum, 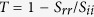, for a wide frequency range can be directly obtained from a single measurement. In addition, to adjust the performance of the metamaterial, a special structure is designed to fix the membranes between adjacent units of the metamaterial, as indicated in Fig. 2(a). A membrane covers the opening of one unit, where the margin of the pipe stretches out of the flange. A thin and light circular gasket is set up around the opening of the pipe and is pressed onto the stretched membrane by two electromagnets (HCN, E4-13/30TH). The stators of the electromagnets are fixed on the flange of the adjacent unit, and the movers touch the circular gasket, which can exert a force on the membrane. The electromagnets controlling the membranes are parallel connected to a DC voltage source. Then, by supplying a voltage to the electromagnets, the movers are promoted by the electromagnetic force and press the circular gasket, which produce additional tension in the membrane and change the impedance and performance of the membrane. By changing the driving DC voltage supplied to the electromagnets, the performance under different driving voltages can be measured with the apparatus.

In addition, an apparatus is established to measure the stiffness of the membranes on the basis of the principle used in a passive metamaterial^13^,^27^. The primary part of the apparatus is similar to one unit of the tunable metamaterial equipped with a membrane and two electromagnets while no side hole is set up. Water is injected into the tube, and the deformation of the membrane induced by the water is measured with a laser ranging sensor (Casati Technologies, PT5028). Repeating the process for several times, the stiffness of the membrane can be obtained from the linear relation between the mass of injected water and the deformation of the membrane. Then, changing the driving voltages supplied to the electromagnets, we can obtain the relation between the stiffness of the membrane and the driving voltage.

To measure the phase velocity in the metamaterial, the exciting signal must be properly chosen. Generally, a sinusoidal signal enveloped by a Gaussian pulse could be adopted to measured phase velocities^44^, while in our system, due to the periodic structure, a Gaussian pulse reflects between the adjacent scatterers and the input and reflected signal overlap with each other, thus it is impossible to determine the phase velocity from the signals obtained at two detection points. Therefore, we use a quasi- sinusoidal signal containing the wave front, which is defined as Sommerfeld precursor^45^,^46^, as the exciting signal. As indicated in Fig. 4a, we count 25 periods from the Sommerfeld precursor until the signals come to steady states. Then, the phase velocity can be achieved by comparing the zero-crossing points in the twenty-fifth period from the wave fronts of both signals measured at the detection points set up in adjacent units. Using this method, the uncertainty induced by the periodicity of a sinusoidal signal can be avoided in the measuring of phase velocities of the metamaterial. It can be observed that although the signal detected in the (N + 1)-th unit precedes that obtained in the N-th unit, which exhibits a negative phase velocity, the Sommerfeld precursor of the former lags that of the latter. Since the transmission velocity of information should be determined by the velocity of the Sommerfeld precursor^45^, the negative phase velocity in this metamaterial does not violate causality indicating that the cause of an even precedes the effect.

To measure the response or recovery time of the metamaterial, a continuous sinusoidal signals is adopted to excite the loudspeaker and the acoustic pressure are measured with a microphone set up in the third unit *U*_3_. From Fig. 5, it can be observed that the amplitudes of the received signals sharply jump with the switching of the driving voltage and then gradually come to a steady state. The recorded signals are processed with STFT to extract the envelope of the variation, as indicated by the red dash lines in Fig. 5(a,b). The relaxation process is fitted with an exponential function, from which the acoustic pressure in the steady state can be achieved, as indicated by *y*_0_ in the inset of Fig. 5. Then, the response or recovery time is defined to be the period when the acoustic pressure changes by 90% from the original value after the jumping to the steady value.

## Additional Information

**How to cite this article**: Chen, Z. *et al*. A tunable acoustic metamaterial with double-negativity driven by electromagnets. *Sci. Rep.* **6**, 30254; doi: 10.1038/srep30254 (2016).

## Figures and Tables

**Table 1:** Transient response times of the tunable metamaterial.

Response Time (s)			Recovery time (s)		
Frequency	4 V	6 V	Frequency	4 V	6 V
470 Hz	18	24	470 Hz	11	11
620 Hz	18	22	620 Hz	11	11
